# Reducing heart failure admission rates in England 2004–2011 are not related to changes in primary care quality: national observational study

**DOI:** 10.1093/eurjhf/hft107

**Published:** 2013-07-11

**Authors:** Rachel Brettell, Michael Soljak, Elizabeth Cecil, Martin R. Cowie, Philippe Tuppin, Azeem Majeed

**Affiliations:** 1 Department of Primary Care Health Sciences, University of Oxford, UK; 2 Department of Primary Care & Public Health, Imperial College London, UK; 3 Royal Brompton Hospital and Imperial College London, UK; 4 Caisse Nationale d'Assurance Maladie des Travailleurs Salariés (CNAMTS), France

**Keywords:** Heart failure, Epidemiology, Hospital admission, Healthcare quality

## Abstract

**Aims:**

Heart failure (HF) is an important clinical problem. Expert consensus has defined HF as a primary care-sensitive condition for which the risk of unplanned admissions may be reduced by high quality primary care, but there is little supporting evidence. We analysed time trends in HF admission rates in England and risk and protective factors for admission.

**Methods and results:**

We used Hospital Episodes Statistics to produce indirectly standardized HF admission counts by general practice for 2004–2011. Clustered negative binomial regression analysis produced admission risk ratios and assessed the significance of potential explanatory covariates. These included population factors (deprivation; HF, coronary heart disease, and smoking prevalence), primary care resourcing [access; general practitioner (GP) supply], and primary care quality (‘Quality and Outcomes Framework’ indicator.) There were 327 756 HF admissions of patients registered with 8405 practices over the study period. There was a significant reduction in admissions over time, from 6.96/100 000 in 2004 to 5.60/100 000 in 2010 (*P* < 0.001). Deprivation and HF prevalence were risk factors for admission. GP supply and access protected against admission. However, these effects were small and did not explain the large and highly significant annual trend in falling admission rates.

**Conclusions:**

The observed fall in admissions over time cannot be explained by the primary care covariates we included. This analysis suggests that the potential for further significant reduction in emergency HF admissions by improving clinical quality of primary care (as currently measured) may be limited. Further work is required to identify the reasons for the reduction in admissions.

## Introduction

Heart failure (HF) is a complex clinical syndrome of symptoms and signs caused by structural or functional impairment of the heart, resulting in impaired pumping efficiency. It affects about900 000 people in the UK,^1^ and >23 million worldwide,^2^ reduces quality of life, and carries a poor prognosis for patients.^3^ High numbers of patients require hospital admission each year.^4^ Furthermore, treatment is costly to society, consuming ∼2% of healthcare budgets annually.^5^ HF is the only major cardiovascular disease which has been increasing in prevalence over time.^6^

HF is considered to be a primary care-sensitive condition (PCSC). PCSCs, also known as ambulatory care-sensitive conditions, are diseases where, according to expert consensus, improving the quality of primary care may reduce the risk of emergency admissions. PCSC lists have been produced by the US and UK governments and by the Organisation for Economic Co-operation & Development (OECD).^7–9^ Population factors, access to primary care, and the quality of primary care services may all affect HF admission rates, but there is little previous research on these associations.

This study aimed to investigate trends in overall HF admissions in England over a 7 year period between 2004–5 and 2010–11. It also examines associations between HF admissions and population factors [deprivation, race, smoking and coronary heart disease (CHD) prevalence]; primary healthcare factors [resourcing, including practice size and general practitioner (GP) supply, and access] and quality of primary care, using indicators from the Quality and Outcomes Framework (QOF), the UK pay-for-performance programme for general practice.^10^

## Methods

### Study design

This was a national observational study of the English population registered with GP practices over a 7 year period (2004–5 to 2010–11).

### Data sources

#### Hospital Episodes Statistics

The Hospital Episode Statistics (HES) database contains hospital admission data from all National Health Service- (NHS) funded hospitals in England. Admissions are coded using the World Health Organization International Classification of Diseases 10th revision (ICD-10.) ICD-10 codes for HF as a primary diagnosis included in the analysis are listed in *Table 1*, in line with the US, UK, and OECD PCSC definitions. This enabled us to explore HF as the main reason for admission, rather than overall HF disease burden in the population. We calculated HF admission data at general practice level for each year of the study to create an observed admissions count for each practice. PCSC data are regularly produced as overall rates to illustrate the overall burden of a disease on health services, so we did not separate first and subsequent admissions.

**Table 1:** Classification of Diseases 10th revision heart failure diagnostic categories as used in primary care-sensitive condition definitions

ICD-10 4 character codes	I11.0 Hypertensive heart disease with (congestive) heart failure
	I13.0 Hypertensive heart and renal disease with (congestive) heart failure
	I13.2 Hypertensive heart and renal disease with both (congestive) heart failure and renal failure
	150.0 Congestive heart failure
	150.1 Left ventricular failure
	150.9 Heart failure, unspecified
	J81X Pulmonary oedema

### Populations

Annual age/sex breakdowns of practice populations obtained from the NHS Information Centre were used to produce indirectly standardized expected rates and counts for HF admissions in each practice. Practices with <500 registered patients (109 of 8405) were excluded from analysis as these were more likely to serve atypical patient populations or to deliver non-standard primary care services. Ethnic breakdowns of HES data were used to produce proportions of patients within each ethnic group in each general practice to enable us to adjust for this in our analysis. This assumes that coding of ethnicity within HES data is representative of practice populations, a previously externally validated method.^11^ We used the Index of Multiple Deprivation (IMD) to adjust for deprivation.^12^ Resident-based IMD scores for 2004, 2007, and 2010 for each Lower Level Super Output Area (LLSOA) were converted to practice-registered population scores using a practice/LLSOA lookup table. We produced smoking prevalence estimates by practice from Office for National Statistics Integrated Household Survey data.

#### Quality and Outcomes Framework and Patient Experience data

The sum of the practice list sizes for the practices included in the QOF represents >99% of registered patients in England. Practices score QOF points based on achievement against multiple indicators within four domains. QOF aims to incentivize and reward good practice, and results are published annually. We used 5 years (2006–7 to 2010–11) of practice-level QOF data from the NHS Information Centre. We used QOF HF prevalence data (from which clinical indicator denominators are drawn) to adjust for HF prevalence.

Particular clinical indicators within QOF change over time, and the 2010–11 HF QOF indicators are displayed in *Table 2*. These are in accordance with UK guidelines released by the National Institute for Health and Clinical Excellence (NICE).^13^ The indicators HF1 and HF2 concern record keeping and initial diagnosis, which are less relevant to ongoing management of HF. However we show data for HF2 as an aid to interpreting HF prevalence data. HF4 has only been included in QOF since 2010–11, so fewer data were available for analysis. We therefore used the HF3 indicator, the percentage of patients with a current diagnosis of HF due to LV dysfunction (LVD) who are currently treated with an ACE inhibitor or an ARB who can tolerate therapy and for whom there is no contraindication, as a marker of how well practices manage HF.

**Table 2:** Relevant quality and outcomes framework indicators

Heart failure indicators	Points	Payment thresholds^a^
Prevalence		
HF prevalence per 100 practice population	N/A	N/A
Records		
HF1: the practice can produce a register of patients with heart failure	4	
Initial diagnosis		
HF2: the percentage of patients with a diagnosis of heart failure (diagnosed after 1 April 2006) which has been confirmed by an echocardiogram or by specialist assessment	6	50–90%
Ongoing management		
HF3: the percentage of patients with a current diagnosis of heart failure due to LVD who are currently treated with an ACE inhibitor or an ARB who can tolerate therapy for whom there is no contraindication.	10	45–80%
HF4: the percentage of patients with a current diagnosis of heart failure due to LVD who are currently treated with an ACE inhibitor or ARB who are additionally treated with a beta-blocker licensed for heart failure or recorded as intolerant to or having a contraindication to beta-blockers.	9	40–65%
Patient experience indicators		
PE07 Patient experience of access (i): percentage of patients who, in the national survery, indicate that they were able to obtain a consultation with their GP	23.5	70–90%
PE08 Patient experience of access (2): percentage of patients who, in the national survery, indicate that they were able to book an appointment with their GP >2 days ahead	35	60–90%

GP, general practitioner, LVD, left ventricular dysfunction.

^a^Where there are two values, these represent the upper and lower achievement levels required to receive the minimum and maximum payment.

To measure access to primary care from patients' perspective, which may be important in preventing emergency admissions from exacerbations, we used indicators PE07, experience of being able to access a GP consultation within 2 days, and PE08, ability to book an appointment >2 days ahead, from QOF.

#### Primary healthcare supply

We obtained data on GP full-time equivalents (FTEs) per 100 000 practice patient population and total practice populations (list size) from the NHS Information Centre.

### Statistical analysis

Negative binomial regression analysis was performed rather than Poisson regression due to overdispersion of the data. This produced incidence rate ratios (IRRs) which in this case are admission rate ratios. Bivariate analysis was performed initially, followed by multivariate analysis. Covariates were selected using backwards stepwise selection, and non-significant factors were removed using likelihood ratio tests. Due to lack of independence, the clustering effect of GP practice was adjusted for in the model. Because we used robust standard errors, which are forced by the use of cluster, the standard Wald test was used to evaluate the model goodness-of-fit. Stata™ version 11 was used for all statistical analysis.

### Ethical approval

This study was a secondary analysis of national data and therefore was not submitted for ethics approval. We had approval from the NHS Information Centre to use HES data for research.

## Results

There were a total of 327 756 admissions due to HF over the 7-year study period from 8405 GP practices. Characteristics of practice populations are shown in *Table 3*. Of note, absolute achievement on the QOF HF3 indicator (number of patients with HF on an ACE inhibitor or ARB) was high, with a median of 90.7% [interquartile range (IQR) 86–100%].

**Table 3:** Characteristics of practice populations and years from which data were analysed

	Mean	Median	IQR	Range	Year(s)^a^
Observed HF admissions/100 000 population	5.66	4	1–8	0–56	2004–2010
Expected (indirectly standardized) HF admissions/100 000 population^b^	5.66	5.55	2.38–7.96	0.02–42.83	2004–2010
Population covariates					
Heart failure prevalence	0.742%	0.7%	0.42–0.92%	0–21.4%	2006–2010
CHD prevalence	5.71%	5.72%	4.72–6.68%	0–14.74%	2010
Smoking prevalence	21.00%	20.96%	16.42–25.77%	0–51.21%	2010
IMD weighting^c^	23.52	21.37	12.43–31.56	2.49–68.97	2004, 2007, 2010
White population (%)	71.09%	77.15%	64.07–84.72%	0.31–100%	2010
Asian population (%)	6.57%	1.27%	0.33–5.40%	0–93.03%	2010
Other population (%)	1.94%	0.73%	0.32–2.26%	0–39.51%	2010
Mixed population (%)	1.00%	0.56%	0.24–1.36%	0–27.71%	2010
Ethnicity data missing (%)	15.68%	13.78%	9.99–19.31%	0–93.90%	2010
Primary care covariates					
QOF HF indicator attainment (HF2)^d^	95.5%	100%	93.8–100%	0–100%	2006–2010
QOF HF indicator attainment (HF3)^e^	90.22%	91.36%	87.64–95.54%	0–100%	2006–2010
PE07 attainment^f^	82.75%	85.39%	77.25–91.79%	0–100%	2008–2010
PE08 attainment^g^	75.68%	78.57%	65.96–88.57%	0–100%	2008–2010
GP FTEs/100 000 patient population	3.55	3	1.72–4.92	0.315–21.88	2004–2010
List size	6488.91	5673	3294–8892	501–40 228	2004–2010

CHD, coronary heart disease; FTE, full-time equivalent; GP, general practitioner; HF, heart failure; IMD, Index of Multiple Deprivation; IQR interquartile range; QOF, Quality and Outcomes Framework.

^a^Years from which data were available for analysis.

^b^Admissions are adjusted for age and sex (indirectly standardized), calculated as [standardized admission ratio (observed admission counts/expected admission counts)] × [national admission rate/100 000].

^c^Weighting for each practice produced by aggregating IMD scores from postcodes of individual registered patients.

^d^HF2: percentage of patients with a diagnosis of HF which has been confirmed by echocardiogram or specialist assessment (since 2006–7).

^e^HF3: percentage of patients with a current diagnosis of HF due to LV dysfunction who are currently treated with an ACE inhibitor or ARB, who can tolerate therapy, and for whom there is no contraindication.

^f^PE07: percentage of patients who, in the national survey, indicate that they were able to obtain a consultation with their GP.

^g^PE08: percentage of patients who, in the national survey, indicate that they were able to book an appointment with their GP >2 days ahead.

**Table 4:** Change in key variables between 2004 and 2010

	2004^a^		2010^b^		% change	*P*-value^c^
	Mean	IQR	Mean	IQR		
Observed admissions/100 000 population	6.96	3–10	5.06	1–8	–27.30	<0.001
Expected admissions/100 000 population^d^	5.31	2.31–7.43	6.00	2.46–8.50	12.99	<0.001
Covariates						
Heart failure prevalence	0.77	0.51–0.97	0.71	0.50–0.90	–7.79	<0.001
QOF HF indicator attainment (HF3)^e^	91.19	86–100	90.68	86–100	–1.41	0.029
PE07 attainment^f^	83.94	79.29–92.98	82.18	76.42–91.00	–2.10	<0.001
PE08 attainment^g^	74.80	64.89–87.61	75.34	66.03–87.50	0.72	<0.001
GP FTEs/100 000 pateint population	3.31	1.6–4.6	3.76	2–5	13.6	<0.001
List size	6248.57	3141–8540	6697.58	2239–9197	7.19	<0.001
IMD weighting	23.84	13.36–32.43	23.75	13.63–32.25	–0.38	0.504

FTE, full-time equivalent; GP, general practitioner; HF, heart failure; IMD, Index of Multiple Deprivation; IQR interquartile range; QOF, Quality and Outcomes Framework.

^a^2004 or first year for which unique data were available for analysis.

^b^2010 or last year for which unique data werr available for analysis.

^c^Paired *t*-test for differences between means.

^d^Admissions are adjusted for age and sex (indirectly standardized), calculated as [standardized admission ratio (observed admission counts/expected admission counts)] × [national admission rate/100 000].

^e^HF3: percentage of patients with a current diagnosis of heart failure due to LV dysfunction who are currently treated with an ACE inhibitor or ARB, who can tolerate therapy, and for whom there is no contraindication.

^f^PE07: percentage of patients who, in the national survey, indicate that they were able to obtain a consultation with their GP.

^g^PE08: percentage of patients who, in the national survey, indicate that they were able to book an appointment with their GP >2 days ahead.

**Figure 1:**
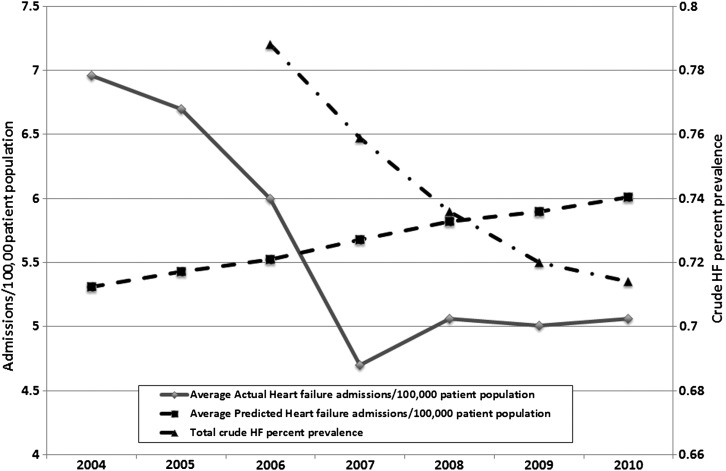
Average heart failure (HF) admission rate/100 000 population compared with the predicted HF admission rate, 2004–5 to 2010–11.

During the study period, average HF admissions per 100 000 patient population fell significantly by 27.3% (*P* < 0.001, paired *t*-test used to assess significance), from 6.96/100 000 in 2004 to 5.06/100 000 in 2010. In contrast, expected HF admissions, based on changes in population demography over the study period, increased by 13% (*P* < 0.001), from 5.31/100 000 in 2004 to 6.00/100 000 in 2010 (see *Figure 1*).

The practice-reported prevalence of HF fell by 7.79% (*P* < 0.001) over the study period, from 0.77/100 000 in 2006 to 0.71/100 000 in 2010. There were also significant changes over time in other key covariates, including markers of primary care quality (see *Table 4*). Considering QOF achievement, achievement of HF2 (percentage of patients with a diagnosis of HF which has been confirmed by echocardiogram or specialist assessment) fell slightly from 97.3% in 2006–7 to 93.8% in 2010–11. There was a small but significant increase of 0.72% (*P* < 0.001) in achievement on the PE08 indicator (percentage of patients who were able to book an appointment with their GP >2 days ahead). However, there was a fall in QOF achievement for the HF3 indicator (number of patients with HF on an ACE inhibitor or ARB) and the PE07 indicator (percentage of patients who indicate that they were able to obtain a consultation with their GP).

*Table 5* shows the total number of observations analysed for each covariate. Where data were not available for each year, data for the closest available year were used as a surrogate. The total number of unique observations for each covariate is shown in parentheses. *Table 5* also shows the results of the bivariate clustered binomial multivariate regression analysis. All covariates retained significance following bivariate analysis; therefore, all were included in the initial multivariate model.

**Table 5:** Bivariate clustered negative binomial regression analysis for heart failure admissions

	No. of observations^a^	IRR	*P*-value	95% CI
Deprivation	56 318 (24 140)	1.017	<0.001	1.016–1.018
Heart failure prevalence	57,811	1.026	0.008	1.007–1.045
CHD prevalence	55,394	1.008	0.001	1.003–1.013
Smoking prevalence	56,263	1.013	<0.001	1.012–1.014
QOF attainment (HF3)^b^	57 022 (40 735)	1.001	0.041	1.000–1.002
PE07 attainment^c^	56 472 (24 208)	0.994	<0.001	0.994–0.995
PE08 attainment^d^	56 044 (24 170)	0.995	<0.001	0.995–0.996
GP supply (FTEs)	57 139 (49 188)	0.963	<0.001	0.961–0.966
Practice list size	57 902	1.000	<0.001	1.000–1.000
2005 (vs. 2004)	57 902	0.952	<0.001	0.929–0.976
2006 (vs. 2004)		0.839	<0.001	0.818–0.860
2007 (vs. 2004)		0.649	<0.001	0.633–0.666
2008 (vs. 2004)		0.659	<0.001	0.642–0.676
2009 (vs. 2004)		0.648	<0.001	0.631–0.664
2010 (vs. 2004)		0.647	<0.001	0.631–0.664

CHD, coronary heart disease; CI, confidence interval; FTE, full-time equivalent; GP, general practitioner; HR, hazard ratio; IRR, incidence rate rato; QOF, Quality and Outcomes Framework.

^a^Where data were not available for each year, data for the closest available year were used as a surrogate. The total number of unique observations is shown in parentheses.

^b^QOF attainment (HF3): percentage of patients with a current diagnosis of heart failure due to LV dysfunction who are currently treated with an ACE inhibitor or ARB, who can tolerate therapy, and for whom there is no contraindication.

^c^PE07 attainment: percentage of patients who, in the national patient survey, indicate that they were able to obtain a consultation with their GP.

^d^PE08 attainment: percentage of patients who, in the national survey, indicate that they were able to book an appointment with their GP >2 days ahead.

*Table 6* shows the results of the multivariate clustered negative binomial regression analysis. The covariate practice list size was dropped from the model in this process as non-significant.

**Table 6:** Multivariate clustered negative binomial regression analysis for heart failure admissions

	IRR	*P*-value	95% CI
Deprivation	1.014367	<0.001	1.013–1.016
Heart failure prevalence	1.072211	<0.001	1.049–1.096
CHD prevalence	0.975838	<0.001	0.967–0.985
Smoking prevalence	1.000494	0.593	0.999–1.002
QOF attainment (HF3)^a^	0.999751	0.625	0.999–1.001
PE07 attainment^b^	0.998424	<0.001	0.998–0.999
PE08 attainment^c^	0.998182	<0.001	0.998–0.999
GP supply (FTEs)	0.99092	<0.001	0.986–0.996
Practice list size	Dropped		
2005 (vs. 2004)	0.950908	<0.001	0.937–0.965
2006 (vs. 2004)	0.831556	<0.001	0.818–0.846
2007 (vs. 2004)	0.646906	<0.001	0.634–0.661
2008 (vs. 2004)	0.664165	<0.001	0.650–0.678
2009 (vs. 2004)	0.651721	<0.001	0.638–0.665
2010 (vs. 2004)	0.650321	<0.001	0.637–0.664

CHD, coronary heart disease; CI, confidence interval; FTE, full-time equivalent; GP, general practitioner; IRR, incidence rate rato; QOF, Quality and Outcomes Framework.

Wald χ^2^(14) = 5284.74.

Log pseudolikelihood = –140815.22.

Probability > χ^2^ = < 0.001.

^a^QOF attainment (HF3): percentage of patients with a current diagnosis of heart failure due to LV dysfunction who are currently treated with an ACE inhibitor or ARB, who can tolerate therapy, and for whom there is no contraindication.

^b^PE07 attainment: percentage of patients who, in the national patient survey, indicate that they were able to obtain a consultation with their GP.

^c^PE08 attainment: percentage of patients who, in the national survey, indicate that they were able to book an appointment with their GP >2 days ahead.

Increasing deprivation score and practice HF prevalence are associated with increased risk of admission. Conversely, GP supply is associated with a reduced risk of admission. However, whilst these effect sizes are significant, they are generally small. Effect sizes are shown as IRRs (in this context, admission risk ratios) and, for example, HF prevalence carries an IRR of 1.07 which represents a 7.2% increase in the admission rate for every percentage point increase in HF prevalence. Of note, the IRRs for markers of primary care supply and quality were particularly small; the IRR for GP supply was 0.991 (i.e. 0.9% reduction in admission rate for each extra GP FTE/100 000 population) and the IRR for PE07 and PE08 QOF indicators was 0.998 (i.e. 0.2% reduction in admission rate for every percentage increase in score on the QOF patient experience indicators). QOF attainment on the HF3 indicator did not significantly affect admission risk, nor did smoking prevalence.

By far the largest effect size on admission risk is seen by year. Year shows strong evidence of progressive protection against admission, with the IRR falling sharply over time. There was a 35% admission risk reduction between 2004 and 2010 (IRR difference 0.650, *P* < 0.0001). This effect retained significance despite adjusting for all the other covariates included in our model, including all our markers of primary care quality, and the effect size changed little after 2006.

## Discussion

We found a significant reduction (27.3%) in total HF admissions over the study period, after adjustment for population factors. This was despite a 13% increase in the expected number of HF admissions based on changes in population demography (see *Figure 1*). This reduction in HF admissions is in contrast to some previous studies which have shown increasing hospitalization rates for HF over time.^14^ On the other hand, other English and Scottish studies have reported an admission peak in 1993–94,^15,^^16^ a recent American study reported a 29.5% reduction in total HF admission rates from 1998 to 2007,^17^ and a Canadian study^18^ also reported a 27.2% reduction from 1994 to 2004. Several studies have also found reductions in the rates of first HF admissions,^4^^,^^19^^,^^20^ although this was not always accompanied by a reduction in overall admissions. As our main focus was on the burden of HF admissions on health services, we did not discriminate between first time and recurrent admissions.

We also report a reduction in HF prevalence of 7.79% between 2004 and 2010. Despite a lack of accurate data for HF prevalence,^21^ the majority of previous studies have suggested that the prevalence of HF is increasing,^22^ possibly because of population ageing, increased diagnosis of HF, and improvements in treatment and survival from ischaemic heart disease (IHD). However, more recent studies have shown a slowing of the rate of increase of HF prevalence in developed countries as the incidence and mortality of HF stabilize.^23,^^24^ The quality of diagnosis has changed little, with the percentage of patients with a diagnosis of HF confirmed by echocardiogram or specialist assessment at 97.3% in 2006–7 and 95.5% in 2010–11; therefore, the fall cannot be explained by patients with incorrect diagnoses being removed. However, even if the prevalence of HF in England is falling, the effect of HF prevalence on admission risk was small and could not fully explain the corresponding fall in HF admissions seen over the study period.

This is the first study to go on to explore the reasons behind time trends in HF admission rates, and access to primary care or the quality of primary care services. Time was progressively protective against admission and had a much larger effect on admission risk than other variables; therefore, the observed fall in HF admissions over time cannot be explained completely by other covariates we considered in our model, including a range of markers of primary care quality. This suggests that the potential for significant further reductions in emergency HF admissions by improving the clinical quality of primary care, as currently measured, may be quite limited.

The QOF attainment on the HF3 indicator did not significantly affect admission rates. This finding is surprising, as ACE inhibitors/ARBs are indicated as first-line HF treatment and have been shown conclusively to reduce mortality and hospitalizations for HF in major clinical trials.^25^ This finding may be explained by the fact that overall scores on the HF3 indicator were generally high throughout the study period, and this ‘ceiling effect’ of QOF achievement may have limited its effectiveness as a discriminator between practices. Practices can also exclude a small proportion of cases from QOF indicators. If the proportion of excluded patients had greatly decreased, and they are now receiving treatment, this could explain some decrease in admissions. However, this proportion has remained steady at 8.1% between 2005–6 and 2010–11. Furthermore, the HF3 QOF indicator does not consider what dose of medication the patients are taking, nor their compliance with prescribed medication. There may be significant variation between the willingness of practices to up-titrate medication to the maximum tolerated dose and to ensure maximum patient concordance, which this analysis was unable to measure.

The HF3 indicator only includes patients with LVD, despite the fact the HF admission totals included both patients with LVD and those with preserved LVEF. Increased use of evidence-based medications may only be expected to have a limited effect in the group with preserved LVEF, and this could at least partly explain why scores on the HF3 indicator were not seen to have a significant effect on admission rates. The non-significance of the HF3 indicator scores may also be related to the ICD-10 code J81X (pulmonary oedema) included as an indicator of an admission for HF. This code was included in the analysis in line with PCSC definitions.^7,^^9^ However, other non-HF diagnoses are potentially included in this diagnostic code (e.g. fluid overload in a dialysis patient). There are few data available regarding the sensitivity or specificity of this code as an indicator of HF.

HF3 was considered the best measurement of ongoing management of HF, and therefore most relevant to HF as a PCSC. HF2, the percentage of patients with a diagnosis of HF which has been confirmed by echocardiogram or specialist assessment, may provide a marker of the quality of HF diagnosis, but achievement has been very high—greater than 95%—throughout the period studied. Similarly, HF4, the percentage of patients who are additionally treated with a beta-blocker, may provide a more sensitive marker of the practice pharmacological management of HF. Beta-blocker uptake has increased in the English population over the study period,^23^ and this could at least partially explain the fall in admission rates. Both these alternative OQF HF indicators could be investigated further in future studies.

We did not include other QOF clinical domain indicators in our analysis, e.g. for IHD, hypertension, diabetes, or smoking, nor did we look at time trends in IHD or smoking prevalence over the study period. However, smoking prevalence was not significantly associated with HF admissions, and IHD prevalence was only associated with a small reduction in admission risk overall, so it is unlikely that these factors could account for the total reduction in admission risk with time. Nonetheless, further investigation of these indicators may be warranted.

Higher use of IHD and hypertension secondary prevention therapies may also contribute to the as yet unexplained reduction in HF admission risk over time. Other pharmacological therapies have also been shown to reduce the risk of admission in HF (as well as HF mortality and other markers of morbidity). These include beta-blockers, aldosterone antagonists, and statins.^26^ Other evidence-based, non-pharmacological strategies are likely to have played a role in reducing admission rates, including specialist HF clinics,^27^ CRT,^28^ specialized multidisciplinary follow-up,^29^ exercise-based rehabilitation,^30^ telemedicine,^31^ specialist nurses,^32^ and self-management programmes.^33^

Other possible reasons to explain the reduction in HF admissions over time could include a general shift away from inpatient care, with limited inpatient bed resources, targets for the reduction of costly hospital admissions, and greater emphasis on community care, including use of risk stratification (e.g. using BNP measurement), rapid follow-up, and more hospital to community HF nursing teams. It is difficult to obtain any national data on these fairly new services, and we were not able to include in the analysis the availability of community care, or markers of access to secondary care. These may warrant further investigation.

Patient perceptions of access to GPs could affect the admission rate by resulting in patients being more likely to attend hospital Accident & Emergency departments and hence be admitted. Patient-reported access to GPs (as measured via the PE07 and PE08 QOF indicators) had a small but significant effect on the risk of admission. Access to community HF teams and rapid follow-up have been shown in randomized controlled trials to reduce the risk of readmission significantly, and may well have a much larger effect on reducing admission risk than access to GPs alone. The fact that the QOF HF3 score was not associated with HF admission risk is not likely to be due to reporting bias, as QOF data are extracted directly from patients' electronic health records, which are used for clinical care. Other factors, including unmeasured patient characteristics, or a practice's ability to ‘game’ the system, have been proposed as reasons why measurements such as QOF may not accurately reflect the quality of clinical care given or received. Conversely, effective, holistic management of HF patients by high quality primary care teams could reduce admissions by influencing medication compliance, self-care, symptom recognition, and consulting behaviour. This is not measured by QOF indicators.

Heart failure is a condition associated with many co-morbidities, and so patients may benefit from having their other diseases controlled. For example, depression is prevalent amongst HF patients and is associated with an increased risk of mortality and hospital admission,^34^ and we did not assess whether primary care was more effectively addressing co-morbid depression. Likewise, appointment availability (as measured by the PE07 and PE08 QOF indicators) is just one aspect of patients' consulting behaviour. The more nuanced aspects of primary care quality are undoubtedly harder to measure, but may be considerably more sensitive.

### Limitations

This analysis included only admissions where HF was coded as the primary reason for admission, although trends for readmissions for HF are also important, especially with a multimorbid, ageing population. Previous reports have suggested that HF readmission rates can be significantly decreased via specific HF-targeted interventions, and it is possible that the fall in admission risk is largely explained by a reduction in the risk of recurrent admissions in high risk individuals. Furthermore, this approach may have missed some cases where HF may have been an underlying reason for admission but not the primary reason.

Most of the IRRs are close to unity, suggesting a small clinical effect. However considered as a percentage reduction in risk admission, they may in fact be clinically relevant. For example, PE08 has an IRR of 0.998, which means there is a 0.2% reduction in admission rate for every 1% increase in score on the QOF indicator PE08. That means that if a practice scores 10% higher on the PE08 QOF indicator, its admission rate is likely to be 2% lower. Given that the IQR for this particular indicator is >20%, this particular indicator alone could result in a >4% difference in admission risk between practices scoring in the top quarter compared with the bottom quarter of practices. Further exploration using individually linked data may be useful in informing future strategies to reduce admission rates further.^35^ Examining trends in the average duration of inpatient stay would identify whether the fall in total admission has been accompanied by a fall in duration of stay, as reported elsewhere.^15^

## Conclusions

This study has shown that HF admissions in England are decreasing over time. Deprivation and high practice HF prevalence increase the risk of admission for HF, whereas greater GP supply and better access to GPs reduce risk of admission. However, despite statistical significance, these effects are small in clinical terms. Coverage of prescribing of an ACE inhibitor or an ARB does not affect the risk of admission. Overall, year has by far the strongest protective effect against admission, with a steady reduction in admission risk from 2004 to 2010. This reduction cannot be explained by available national markers of primary care quality or access to primary care, and this study does not provide any support for the hypothesis that in the UK HF admissions are sensitive to primary care quality, as currently measured. The reduction may be due to the development of non-pharmacological interventions about which there is a lack of national data, or the more nuanced aspects of primary care quality not measured by QOF. Further work is required to identify the reasons behind the reduction in admissions, for example by longitudinal patient-level analysis of electronic health records, supplemented with local surveys of new HF services, or data from the Heart Failure National Clinical Audit.^36^ This may enable us to identify which initiatives are having a greater impact on admission rates and provide further evidence for improving HF services.

### Funding

The NHS Institute for Improvement and Innovation. They agreed the overall study design but had no further input, and the researchers were independent from the funding body. Imperial College London is also grateful to the NIHR Biomedical Research Centre Scheme and the NIHR Collaboration for Leadership in Applied Health Research and Care scheme. M.R.C.'s salary is supported by the NIHR Biomedical Research Unit at the Royal Brompton hospital.

**Conflict of interest:** none declared

## References

[1] Sutherland K (2010). Bridging the Quality Gap: Heart Failure.

[2] McMurray JJ, Petrie MC, Murdoch DR, Davie AP (1998). Clinical epidemiology of heart failure: public and private health burden. Eur Heart J.

[3] Stewart S, MacIntyre K, Hole DJ, Capewell S, McMurray JJV (2001). More ‘malignant’ than cancer? Five-year survival following a first admission for heart failure. Eur J Heart Fail.

[4] Jhund PS, Macintyre K, Simpson CR, Lewsey JD, Stewart S, Redpath A, Chalmers JWT, Capewell S, McMurray JJV (2009). Long-term trends in first hospitalization for heart failure and subsequent survival between 1986 and 2003: a population study of 5.1 million people. Circulation.

[5] Stewart S, Jenkins A, Buchan S, McGuire A, Capewell S, McMurray JJJV (2002). The current cost of heart failure to the National Health Service in the UK. Eur J Heart Fail.

[6] Owan TE, Hodge DO, Herges RM, Jacobsen SJ, Roger VL, Redfield MM (2006). Trends in prevalence and outcome of heart failure with preserved ejection fraction. N Engl J Med.

[7] Organisation for Economic Cooperation & Development (2011). HCQI Health Promotion, Prevention and Primary Care.

[8] Agency for Healthcare Research & Quality DoHHS (2007). AHRQ Guide to Prevention Quality Indicators (Version 3.1).

[9] Department of Health The NHS Outcomes Framework 2012/13: Technical Appendix.

[10] Information Centre for Health & Social Care (2012). The Quality & Outcomes Framework.

[11] Hull SA, Rivas C, Bobby J, Boomla K, Robson J (2009). Hospital data may be more accurate than census data in estimating the ethnic composition of general practice populations. Inform Prim Care.

[12] Department of Communities & Local Government (2011). English Indices of Deprivation 2010 (revised).

[13] National Institute for Clinical & Public Health Excellence (2010). Chronic Heart Failure: Management of Chronic Heart Failure in Adults in Primary and Secondary Care.

[14] McMurray J, McDonagh T, Morrison CE, Dargie HJ (1993). Trends in hospitalization for heart failure in Scotland 1980–1990. Eur Heart J.

[15] Stewart S, MacIntyre K, MacLeod MMC, Bailey AEM, Capewell S, McMurray JJV (2001). Trends in hospitalization for heart failure in Scotland, 1990–1996. An epidemic that has reached its peak?. Eur Heart J.

[16] Gnani S, Ellis C, Majeed A Trends in hospital admissions and case fatality due to heart failure in England, 1990/91 to 1999/2000. http://www.ons.gov.uk/ons/rel/hsq/health-statistics-quarterly/no-12-winter-2001/index.html.

[17] Chen J, Normand S-LT, Wang Y, Krumholz HM (2011). National and regional trends in heart failure hospitalization and mortality rates for Medicare beneficiaries, 1998–2008. JAMA.

[18] Arnold JM, Liu P, Demers C, Dorian P, Giannetti N, Haddad H, Heckman GA, Howlett JG, Ignaszewski AJDE, Jong P, McKelvie RS, Moe GW, Parker JD, Rao V, Ross HJ, Sequeira EJ, Svendsen AM, Teo K, Tsuyuki RT, White M, Society CC (2006). Canadian Cardiovascular Society consensus conference recommendations on heart failure 2006: diagnosis and management. Can J Cardiol.

[19] Schaufelberger M, Swedberg K, Köster M, Rosén M, Rosengren A (2004). Decreasing one-year mortality and hospitalization rates for heart failure in Sweden; Data from the Swedish Hospital Discharge Registry 1988 to 2000. Eur Heart J.

[20] Wasywich CA, Gamble GD, Whalley GA, Doughty RN (2010). Understanding changing patterns of survival and hospitalization for heart failure over two decades in New Zealand: utility of ‘days alive and out of hospital’ from epidemiological data. Eur J Heart Fail.

[21] Davies M, Hobbs F, Davis R, Kenkre J, Roalfe AK, Hare R, Wosornu D, Lancashire RJ (2001). Prevalence of left-ventricular systolic dysfunction and heart failure in the Echocardiographic Heart of England Screening study: a population based study. Lancet.

[22] Cleland JGF, Swedberg K, Follath F, Komajda M, Cohen-Solal A, Aguilar JC, Dietz R, Gavazzi A, Hobbs R, Korewicki J, Madeira HC, Moiseyev VS, Preda I, van Gilst WH, Widimsky J, Freemantle N, Eastaugh J, Mason J (2003). The EuroHeart Failure survey programme—a survey on the quality of care among patients with heart failure in Europe: Part 1: patient characteristics and diagnosis. Eur Heart J.

[23] Hawkins NM, Scholes S, Bajekal M, Love H, O'Flaherty M, Raine R, Capewell S (2012). Community care in England: reducing socioeconomic inequalities in heart failure. Circulation.

[24] Curtis LH, Whellan DJ, Hammill BG, Hernandez AF, Anstrom KJ, Shea AM, Schulman KA (2008). Incidence and prevalence of heart failure in elderly persons, 1994–2003. Arch Intern Med.

[25] Flather MD, Yusuf S, Køber L, Pfeffer M, Hall A, Murray G, Torp-Pedersen C, Ball S, Pogue J, Moyé L, Braunwald E (2000). Long-term ACE-inhibitor therapy in patients with heart failure or left-ventricular dysfunction: a systematic overview of data from individual patients. Lancet.

[26] Lipinski MJ, Cauthen Ca, Biondi-Zoccai GGL, Abbate A, Vrtovec B, Khan BV, Vetrovec GW (2009). Meta-analysis of randomized controlled trials of statins versus placebo in patients with heart failure. Am J Cardiol.

[27] Thomas R, Huntley A, Mann M, Huws D, Paranjothy S, Elwyn G, Purdy S (2013). Specialist clinics for reducing emergency admissions in patients with heart failure: a systematic review and meta-analysis of randomised controlled trials. Heart.

[28] Lemos Júnior HPd, Atallah AN (2009). Cardiac resynchronization therapy in patients with heart failure: systematic review. São Paulo Med J.

[29] McAlister Fa, Stewart S, Ferrua S, McMurray JJJV (2004). Multidisciplinary strategies for the management of heart failure patients at high risk for admission: a systematic review of randomized trials. J Am Coll Cardiol.

[30] Davies EJ, Moxham T, Rees K, Singh S, Coats AJS, Ebrahim S, Lough F, Taylor RS (2010). Exercise based rehabilitation for heart failure. Cochrane Database Syst Rev.

[31] Inglis SC, Clark RA, McAlister FA, Ball J, Lewinter C, Cullington D, Stewart S, Cleland JGF (2010). Structured telephone support or telemonitoring programmes for patients with chronic heart failure. Cochrane Database Syst Rev.

[32] Stewart S, Blue L, Walker A, Morrison C, Mcmurray JJV (2002). An economic analysis of specialist heart failure nurse management in the U.K,Can we afford not to implement it?. Eur Heart J.

[33] Ditewig JB, Blok H, Havers J, van Veenendaal H (2010). Effectiveness of self-management interventions on mortality, hospital readmissions, chronic heart failure hospitalization rate and quality of life in patients with chronic heart failure: a systematic review. Patient Educ Couns.

[34] Lane DA, Chong AY, Lip GYH (2005). Psychological interventions for depression in heart failure. Cochrane Database Syst Rev.

[35] Gwadry-Sridhar FH, Flintoft V, Lee DS, Lee H, Guyatt GH (2004). A systematic review and meta-analysis of studies comparing readmission rates and mortality rates in patients with heart failure. Arch Intern Med.

[36] National Institute for Cardiovascular Outcomes Research (2012). National Cardiovascular Clinical Audits and their associated registries: Centre for Cardiovascular Preventions and Outcomes, University College London. Heart Failure National Clinical Audit dataset v3.0.

